# Community-Based Participatory Research: A Vehicle to Promote Public Engagement for Environmental Health in China

**DOI:** 10.1289/ehp.11399

**Published:** 2008-06-13

**Authors:** Robbie Ali, Kenneth Olden, Shunqing Xu

**Affiliations:** 1 Graduate School of Public Health, University of Pittsburgh, Pittsburgh, Pennsylvania, USA; 2 Laboratory of Molecular Carcinogenesis, National Institute of Environmental Health Sciences, National Institutes of Health, Department of Health and Human Services, Research Triangle Park, North Carolina, USA; 3 Tongji Medical College, Huazhong University of Science and Technology, Wuhan, People’s Republic of China

**Keywords:** CBPR, China, China environmental health, community-based participatory research, environmental justice, international environmental health

## Abstract

**Background:**

In the past 25 years, China has experienced remarkable economic growth and rapid agricultural-to-industrial and rural-to-urban transitions. As a consequence, China now faces many daunting environmental challenges that are significantly affecting human health and quality of life, including indoor and outdoor air pollution, water pollution, deforestation, loss of agricultural land, and sustainability. Chinese government leaders have recently emphasized the need for better environmental protection practices along with interventions involving strong public participation.

**Objectives:**

Community-based participatory research (CBPR) is a collaborative approach to research that involves community members, organizational representatives, and researchers as equal participants in all phases of the research process. Over the past 15 years, CBPR has gained recognition and acceptance and is now valued as a means to effect change and provide scientific knowledge relevant to human health and the environment. In this article we highlight the success of CBPR in the United States and suggest that it could be a useful model for addressing environmental health problems in the People’s Republic of China.

**Discussion:**

CBPR can reduce the tension between science and society by promoting genuine communication, by enabling scientists and administrators to listen and respond to the public, by allowing communities to help shape the research agenda, and by increasing accountability of researchers and governments to the public.

**Conclusions:**

CBPR can potentially help improve environmental health in China, but it is likely to take a different form than it has in the West because the government will be leading the way.

In the past 25 years, China has experienced remarkable economic growth and rapid agricultural-to-industrial and rural-to-urban transitions. Its gross domestic product (GDP) has increased at rates of 8–9% annually, and hundreds of millions of people have been lifted from extreme poverty. As a consequence of this unprecedented growth, China now faces many daunting environmental challenges that are significantly affecting human health and quality of life. China’s environmental sustainability index is 133 among 145 countries ranked in 2005 (Esty et al. 2005). Because China has the largest population and the third largest land area of any nation, it is significant that environmental management and sustainability are now recognized as a high priority of the government.

Some specific examples of the environmental challenges facing China include indoor and outdoor air pollution; contamination of land from use of water that contains industrial waste, untreated human and animal sewage, and excessive use of fertilizers and pesticides; deforestation; loss of agricultural land; and sustainability. The World Bank (2007) has estimated that 20 of the 30 cities with the most polluted air are in China and that outdoor air pollution from industrial, vehicular, and energy-generating sources causes 350,000–400,000 deaths each year. Water pollution results from unregulated industrial growth, rapid expansion of urban areas with insufficient infrastructure for water treatment, intense use of fertilizers, and irrigation of farmland with water containing untreated sewage and industrial waste. The World Bank (2007) also estimated that 60,000 people each year die prematurely from waterborne infectious diseases. The Chinese Academy of Agricultural Sciences has estimated that pesticide exposure affects the health of 100,000 people annually (Huang et al. 2000), and this may be a significant underestimation of the actual exposure, because approximately one-half of the fruits and vegetables tested from two provinces had levels of carbamate and organophosphate pesticides in excess of government standards [Huang et al. 2000; Yunnan Entomological Society (YES) Newsletter 2001]. Such pesticide exposures are reported to have caused reproductive abnormalities among male farmworkers (Padungtod et al. 1999, 2000). Furthermore, an estimated 12 million tons of grain are contaminated annually with heavy metals, at a cost to the country of US $2.5 billion (Padungtod et al. 2000).

Finally, China’s State Environmental Protection Administration (SEPA) estimated that use of contaminated water, excess fertilizer, solid waste, pesticides, and other pollutants has damaged about 10% (12.3 million hectares) of China’s arable land (China Daily 2006; Reuter’s 2007). Because only about 13% of China’s land area is suitable for farming, pollution damage combined with loss of arable land due to industrial use and urbanization are endangering China’s food security. Also, China’s growing livestock-farming industry produces 2.7 billion tons of waste annually (Padungtod et al. 2000). About 97% of the 20,000 medium- to large-sized livestock farms do not have waste treatment facilities, so waste is discharged directly into the environment, polluting the air, water, and soil (Padungtod et al. 2000).

Reversing the environmental degradation associated with economic growth and other human activities will require strong government leadership and an enormous economic investment in new technologies. For example, the annual economic costs of air and water pollution in China alone have been estimated at 3% and 6% of the GDP, respectively (World Bank 2007), effectively blunting in real terms anywhere from one-third to two-thirds of China’s dramatic economic growth. Moreover, these estimates do not take into account the demands and pressures that such growth has on the country’s natural resources and thus on its ability as a nation to achieve long-term sustainable improvements in quality of life for present and future generations. On the global scale, China is estimated to have the second largest “ecological footprint” of any nation (second only to the United States), and this footprint continues to expand rapidly (Liu and Diamond 2005). Because China’s environmental policies, and their success or failure, will have a significant impact on the global economy, global ecology, and global health, it is critical for both China and the rest of the world to find solutions to these environmental problems.

## Reasons for Optimism

But, in spite of the enormous challenge, there are reasons for optimism that China will be successful in addressing its environmental problems. First, the central government acknowledges these problems and has made environmental protection a high priority. For example, improvement in the environment is now a key element used to evaluate the performance of provincial and municipal leaders. Government leaders (e.g., Premier Wen Jiabao) have recently emphasized the need for better environmental protection practices with the implementation of interventions involving strong public participation (Yue 2006). China’s ever-growing international presence (e.g., membership in the World Trade Organization and the 2008 Olympic Games in Beijing) has provided increased incentives to employ cleaner production practices, improved resource use, and more energy-efficient operations. Because of the increased emphasis on environmental protection, specific environmental advances in China over the past decade are already evident; these include a gradual reduction in urban air pollution, the phasing out of leaded gasoline, and the introduction of land conservation measures such as reforestation programs and a ban on logging. Furthermore, China’s environmental laws, policies, and regulations continue to be developed and are becoming more stringent, and there are efforts to adopt green GDP practices that take into account environmental costs of development.

Both the central government’s call for public participation and our suggestion of the use of community-based participatory research (CBPR) should be highly successful in China because this country has had a long history of government-driven mass public campaigns. Among the most remarkably successful of these has been the schistosomiasis control efforts that began in the 1950s (Wang 2000; Yuan et al. 2002). These consisted of public application of molluscicides, environmental modifications (e.g., drainage of swampy areas), mass chemotherapy, public education, and improvements in sanitation. Also, “grassroots” non–government-driven public participation in environmental issues, although still rare, is beginning to occur. For example, a community-based effort got villagers involved in the cleanup of the Han River in Hubei Province. The high levels of arsenic, chromium, and benzene discharged from upstream industries were thought to be associated with increased risk of various cancers in the village of Zhaiwan. This successful community-initiated activity caught the attention of high-level government officials, who assisted with the remediation effort by providing money for a system of wells and pipes to make safe drinking water available to 803 households (Haggart and Lan 2000, 2006).

In 2006, Pan Yue, the deputy director of the SEPA stressed the need for government accountability and for public involvement in environmental issues, stating that public participation in environmental protection is part of the development process of a socialist democracy (Yue 2006). He also outlined key government actions needed in China to promote public participation in environmental protection, including responding to and protecting the public’s legal right to participate in environmental protection; increasing the transparency of environmental information so that “the force of public opinion can put pressure on those who destroy the environment”; facilitating public participation in environmental decision making through meetings and hearings, legally required for large projects as of 2003; allowing public interest environmental litigation wherein citizens, communities, and government agencies can bring lawsuits on behalf of the wider public; and increasing cooperation with environmental nongovernmental organizations (e.g., providing professional training, creating platforms for public communication, organizing activities, and sponsoring public opinion surveys on particular policies).

## CBPR Model

Given the long history of government, industry, and community collaborations in China, fertile ground and infrastructure already exist for the integration of community-based initiatives in environmental health protection, with the government likely to lead the way in the near term. CBPR emerged in the United States over the past 25 years as an important alternative to more traditional investigator-initiated research that used community residents as research subjects and, at times, created a sense of inequity, mistrust, and exploitation (Israel et al. 1998; Northridge et al. 2000; O’Fallon and Dearry 2001; O’Fallon et al. 2003; Olden 1998; Olden et al. 2001). CBPR has been described as research “with” and “for” communities, rather than research “on” communities. It is a comprehensive approach to public health practice and research on the social and environmental determinants of health. Its aim is to increase knowledge and understanding of a given phenomenon and to translate the knowledge gained into action-oriented interventions to achieve behavioral and policy change to improve public health and quality of life for community members (O’Fallon et al. 2003).

CBPR is a collaborative approach to research that involves community members, organizational representatives, and researchers as equal participants in all phases of the research process. This process includes identifying health issues and choosing research topics of concern to the community; developing assessment tools; collecting, analyzing, and interpreting data; determining how data can be used to inform decision making; developing the research design; designing, implementing, and evaluating interventions; interpreting, disseminating, and translating findings into policies and programs; and monitoring of social and ethical concerns. In CBPR, all partners contribute their respective expertise and share responsibilities and power of decision making (Israel et al. 1998; Northridge et al. 2000; O’Fallon and Dearry 2001; O’Fallon et al. 2003; Olden 1998; Olden et al. 2001).

Community-based and community-oriented approaches to research developed in many instances as researchers and agencies attempted to respond to concerns raised by the general public or by citizen groups about specific health issues affecting local communities or exposed groups of people (Wallerstein and Duran 2003). CBPR has now been endorsed as a valuable research approach in the United States by the National Institutes of Health, the Centers for Disease Control and Prevention (CDC), and the Institute of Medicine (1999). Since the early 1990s, CBPR has been a large part of the translational research program of the National Institute of Environmental Health Sciences (NIEHS), specifically in the Environmental Justice Program, a program developed to address the immediate environmental health concerns of socioeconomically disadvantaged communities throughout the United States. The objective of this program is to determine whether living and working in the most polluted environments contributes to the disparities in health experienced by this population. By involving the community, the lessons learned can be effectively translated into effective public health prevention measures without the long delay characteristic of traditional investigator-driven research. The Agency for Toxic Substances and Disease Registry has also promoted a CBPR approach to environmental health issues (Sexton et al. 1993).

Despite the challenges to conducting CBPR described in the literature (Cohall 1999; Israel et al. 1998), this research approach is beginning to develop a track record of success because the various research groups targeted indicators that were both actionable and changeable. For example, the University of Iowa worked with the concentrated animal feeding operations in the state to develop a better way to protect air and water quality threatened by the release of ammonia and hydrogen sulfide from hog waste (Merchant J, personal communication). Many of the recommendations generated by this report became established policies of the state government to protect the environment and quality of life of residents from waste generated by hog farms. Similarly, a CBPR effort in Oklahoma mobilized a Native American community to prevent heavy metal contamination of their land from the mining of lead and zinc (Kegler and Malcoe 2004).

A partnership between the community and the University of Cincinnati in Ohio examined the efficiency of the city’s dust removal and contaminant control procedures during the demolition of old housing stock (Bornscheim R, personal communication). The control procedures were found to be very inefficient, and dust collected in the community contained high levels of lead. Based on these findings, the Cincinnati Office of Environmental Management developed a new set of regulations to control lead in dust from demolition of old housing (Porras C, personal communication). A community-based organization in San Diego, California, partnered with an environmental health scientist at the University of Southern California to investigate the human health effects of exposure to hexavalent chromium, a toxic air pollutant generated by chrome plating shops located in residential neighborhoods. This collaboration obtained data to demonstrate that exposure under these circumstances exceeded regulatory limits. As a consequence, the municipal government closed the chrome plating industry in residential communities (Farbis and Baker 2002; O’Fallon et al. 2003). Another CBPR activity was carried out by the University of California, Los Angeles, in a heavily industrialized Hispanic community in southeastern Los Angeles. The health effects of heavy dust from a broken concrete and asphalt open-air waste site was a major concern of the local residents. Air monitoring in the area surrounding the waste site confirmed the impression of community residents that the dust contained particulate matter that caused respiratory distress among asthmatics (Porras C, personal communication). This information was used to convince the city government to remediate the concrete/asphalt waste site (Olden 1998). The same CBPR program participants also generated data that led to the removal of an open-air ground glass storage area from the community because glass particulates found in the air caused nose bleeds.

The Environmental Research Center of the University of California, Davis, worked with the agriculture community to develop activities to verify the accuracy of bioassays used by the state government to monitor farmworkers for pesticide toxicity. The university researchers discovered that the commercial bioassays in use were very inaccurate, often underestimating the level of acetylcholinesterase by as much as 40% (Matsumura F, personal communication). Furthermore, the various assays used to assess the degree of pesticide poisoning in various municipalities employed completely different units of measurement, making meaningful comparisons difficult. By working with the state regulatory agency, a new, more reliable test was developed and deployed to bring the fulfillment of the legal requirement in line with the public health intent of the law (Olden 1998).

Members of the West Harlem Environmental Action Committee, a community-based environmental group in New York City, formed a partnership with the Columbia University School of Public Health, the CDC, and the NIEHS to investigate the impact of diesel exhaust from city-operated trucks and buses on the air quality in the West Harlem community (Northridge et al. 1999). This research effort was developed in response to community-initiated concerns, and community members were actively involved in every phase of the study, from formulation of the hypothesis and study design to interpretation and dissemination of the findings. The researchers found that fine particulate matter (≤ 2.5 μm in aerodynamic diameter) and polycyclic aromatic hydrocarbons (PAHs) in the air were directly correlated with the number of diesel trucks and buses passing through the community (Kinney et al. 2000; Northridge et al. 1999). Furthermore, PAHs could be detected in urine of the exposed population, and about 30% had obstruction of the small airways in the lungs that is characteristic of asthma (Northridge et al. 1999). These findings were used to convince the municipal government to begin a phased process of replacing its aging fleet of diesel buses with new ones powered by natural gas.

These examples illustrate ways in which local communities can work with university researchers and government agencies to improve the environment, human health, and quality of life. In addition to the public benefits and policy changes from CBPR, other accrued benefits include capacity building and improvement in the potential for productive employment. Over the past 15 years, CBPR has gained wider recognition and acceptance and is now valued as a means to effect change and to provide scientific knowledge relevant to human health and the environment. For other examples and for more comprehensive treatment of the success of CBPR, we refer readers to Israel et al. (1998), O’Fallon and Dearry (2001), O’Fallon et al. (2003), Northridge et al. (2005), and Wing (2005).

## Context Dependency of CBPR

CBPR in the United States was initiated by government agencies in response to activism by community groups concerned about the impact of the environment on their health. The ultimate success of this effort to involve communities in environmental health research required strong leadership and support of government agencies (Northridge et al. 2005; Olden et al. 2001). Top-down or government-initiated efforts to promote CBPR, without pressure from community groups, could be just as effective in China as the United States model, provided the Chinese government is serious about involving community residents. However, if CBPR is to be introduced in China, it is likely to be context dependent and to take a different form than it has in the West, with the government itself leading the way because of the generally low environmental consciousness among citizens and the fact that independent citizen action is novel and foreign. China’s civil society is underdeveloped, and the few nongovernmental organizations that do exist focus mainly on public awareness rather than action. For these reasons, foremost consideration must be given to the Chinese government’s leadership role in promoting CBPR. The government will likely be cautious in supporting an activity that can potentially lead to criticisms of its environmental protection policies and practices. CBPR would likely bring attention to the true level of transparency of environmental information and of government accountability for environmental stewardship.

## Conclusion

In this article we highlight the success of CBPR in the United States and suggest that it could be a useful model for addressing environmental health problems in the People’s Republic of China. CBPR provides an approach to develop and implement interventions in the context of place, that is, in the context of cultural values, economic status, and other community influences. CBPR emphasizes the relevance, quality, and validity of the research findings and empowers communities to find solutions to their own problems. Most important, it reduces the time between concept, discovery, and translation in terms of policy or behavioral change. Furthermore, communities can build upon the infrastructure created by CBPR activities to refine or test new strategies to promote civic engagement.

Science and technology are important in every aspect of modern society, and the translation of knowledge to improve the human condition requires strong relationships between science and society. Although our capacity to improve human health and quality of life has accelerated over the past 35 years, our ability to translate scientific discoveries into products, goods, and services has not kept pace. The slow pace of translation has created tension and conflict with respect to how priorities are set by the leadership of the biomedical research enterprise. CBPR can reduce the tension between science and society by promoting genuine communication, by listening and responding to the public, by allowing communities to help shape the research agenda, and by increasing accountability of researchers and governments to the public.

## References

[b1-ehp-116-1281] China Daily (2006). One tenth of arable land suffers from pollution.

[b2-ehp-116-1281] Cohall A (1999). Applied prevention research: challenges of working with urban communities. Am J Prevent Med.

[b3-ehp-116-1281] Esty D, Levy M, Srebotnjak T, Sherbinin A (2005). Environmental Sustainability Index: Benchmarking National Environmental Stewardship.

[b4-ehp-116-1281] Farbis P, Baker J (2002). State Confirms Public Health Hazard [Press Release].

[b5-ehp-116-1281] Haggart K, Lan M (2000). Blighted village raises fears about south-north water scheme. Three Gorges Probe, 29 January. http://www.probeinternational.org/catalog/content_fullstory.php?contentId=2725&cat_id=24.

[b6-ehp-116-1281] Haggart K, Lan M (2006). Is Zhaiwan’s pipe dream coming true? Three Gorges Probe, 3 March. http://www.probeinternational.org/catalog/content_fullstory.php?contentId=2994&cat_id=24.

[b7-ehp-116-1281] Huang JK, Qiao FB, Zhang LX, Rozelle S (2000). Farm Pesticide, Rice Production, and Human Health.

[b8-ehp-116-1281] Institute of Medicine (1999). Toward Environmental Justice, Research, Education, and Health Policy Needs.

[b9-ehp-116-1281] Israel BA, Schulz AJ, Parker EA, Becker AB (1998). Review of community-based research: assessing partnership approaches to improve public health. Annu Rev Public Health.

[b10-ehp-116-1281] Kegler M, Malcoe M (2004). Results from a lay health advisor intervention to prevent lead poisoning in Native American children. Am J Public Health.

[b11-ehp-116-1281] Kinney PL, Aggarwal M, Northridge ME, Janssen NAH, Shephard P (2000). Airborne concentrations of PM2.5 and diesel exhaust particles on Harlem sidewalks: a community-based pilot study. Environ Health Perspect.

[b12-ehp-116-1281] Liu J, Diamond J (2005). China’s environment in a globalizing world. Nature.

[b13-ehp-116-1281] Northridge ME, Shoemaker K, Jean-Louis B, Ortiz B, Swaner R, Vaughan RD, Goehl TJ (2005). Using community-based participatory research to ask and answer questions regarding the environment and health. Essays on the Future of Environmental Health Research: A Tribute to Dr. Kenneth Olden.

[b14-ehp-116-1281] Northridge ME, Vallone D, Merzel C, Green D, Shepard P, Cohall AT (2000). The adolescent years: an academic-community partnership in Harlem comes of age. J Public Health Manag Pract.

[b15-ehp-116-1281] Northridge ME, Yankura J, Kinney PL, Santella RM, Shepard P, Riajas Y (1999). Diesel exhaust exposure among adolescents in Harlem: a community-driven study. Am J Public Health.

[b16-ehp-116-1281] Olden K (1998). The complex interaction of poverty, pollution, health status. Scientist.

[b17-ehp-116-1281] Olden K, Guthrie J, Newton SA (2001). A bold new direction for environmental health research. Am J Public Health.

[b18-ehp-116-1281] O’Fallon LR, Dearry A (2001). Commitment of the National Institute of Environmental Health Sciences to community-based participatory research for rural health. Environ Health Perspect.

[b19-ehp-116-1281] O’Fallon LR, Wolfle GM, Brown D, Dearry A, Olden K (2003). Strategies for setting a national research agenda that is responsive to community needs. Environ Health Perspect.

[b20-ehp-116-1281] Padungtod C, Hassold TJ, Millie E, Ryan LM, Savitz DA, Christiani DC (1999). Sperm aneuploidy among Chinese pesticide factory workers: scoring by the FISH method. Am J Ind Med.

[b21-ehp-116-1281] Padungtod C, Savitz DA, Overstreet JW, Christiani DC, Ryan LM, Xu X (2000). Occupational pesticide exposure and semen quality among Chinese workers. J Occup Environ Med.

[b22-ehp-116-1281] Reuter’s (2007). China Says Ten Percent of Farming Land Contaminated.

[b23-ehp-116-1281] Sexton K, Olden K, Johnson BL (1993). Environmental justice: the central role of research in establishing a credible scientific foundation for informed decision making. Toxicol Ind Health.

[b24-ehp-116-1281] Wallerstein N, Duran B, Minkler M, Wallerstein N (2003). The conceptual, historical, and practice roots of community-based participatory research and related participatory traditions. Community-Based Participatory Research for Health.

[b25-ehp-116-1281] Wang R (2000). Critical health literacy: a case study from China in schistosomiasis control. Health Promot Int.

[b26-ehp-116-1281] Wing S, Goehl TJ (2005). Environmental justice, science, and public health. Essays on the Future of Environmental Health Research: A Tribute to Dr. Kenneth Olden.

[b27-ehp-116-1281] World Bank (2007). Cost of Pollution in China: Economic Estimates of Physical Damages.

[b28-ehp-116-1281] Yuan H, Jiang Q, Zhao G, He N (2002). Achievements of schistosomiasis control in China. Mem Inst Oswaldo Cruz Rio de Janeiro.

[b29-ehp-116-1281] Yue P (2006). The environment needs public participation. China Dialogue, 5 December. http://www.chinadialogue.net/article/show/single/en/604-the-environment-needs-public-participation.

[b30-ehp-116-1281] YES (2001). Yunnan Entomological Society Newsletter.

